# Results of an endocrine follow-up of obese pre-diabetic and diabetic patients before and after bariatric surgery, through an evaluation of nutritional and biochemical variables, analyzed 14, 28, 42 and 56 days after surgery at Uberlândia-Mg, Brazil

**DOI:** 10.1186/1758-5996-7-S1-A92

**Published:** 2015-11-11

**Authors:** Mariana Scramin Guimarães Maciel, Ana Luísa Conceição de Jesus, Denise Rosso Tenório Wanderley Rocha, André Rocha Jorge, Alberto Krayyem Arbex

**Affiliations:** 1 IPEMED-instituto de Pesquisa e Ensino Médico, Rio de Janeiro, Brazil

## Background

Obesity and diabetes are chronic metabolic diseases, regarded nowadays as serious public health challenges. The treatment of severe obesity is effectively achieved through bariatric surgery, resulting in a better control of diabetes and metabolic markers of disease.

## Objective

To evaluate the changes in food intake, body composition and biochemical markers among patients during eight-week following Gastric Bypass Surgery Roux-Y (RYGB), in a multidisciplinary approach team.

## Materials and methods

This prospective study included 22 women undergoing RYGB, of which 6 had pre-diabetes, and 1 was diabetic. The team consisted of an endocrinologist, a surgeon, a nutritionist and a psychologist. Anthropometric, nutritional and biochemical parameters were analyzed preoperatively and 14, 28, 42 and 56 days after surgery. Specifically, 4 goals were set: 1) to describe the presence of morbidities associated with obesity, and their evolution after surgery; 2) to study the alterations in the biochemical variables after the RYGB proceeding; 3) to evaluate the nutritional changes related with the pre- and post-surgery times; and 4) to follow the changes in body mass index (BMI) and weight loss during pre- and post-surgery, on 4 different time lapses after surgery.

## Results

After 56 days of surgery, the rate of pre-diabetes and diabetes dropped from 32% to 5% of the patients. An increase in calorie intake was documented from days 14 to 56 after surgery. A greater intake of protein was also noticed, though a lower intake of iron and calcium was also present. Decreases in body weight and BMI were associated with reduced blood levels of total cholesterol, VLDL-C, LDL-C, triglycerides, and glucose. Weight loss and decreased BMI were reported as well. Dyslipidemia was found among 50% of patients before surgery, and only 1 among 22 patients still had dyslipidemia after surgery.

## Conclusions

A significant reduction was documented in the prevalence of prediabetes and diabetes among patients undergoing bariatric surgery, along with an increase in calorie intake after surgery. This study is important for small groups of surgical intervention in obese diabetic and prediabetic patients, in order to better understand approaches towards this high-risk population.

